# Characterizing the Interplay of Lymphocytes in Graves’ Disease

**DOI:** 10.3390/ijms24076835

**Published:** 2023-04-06

**Authors:** Mackenzie Hansen, Abigail Cheever, K. Scott Weber, Kim L. O’Neill

**Affiliations:** Department of Microbiology and Molecular Biology, Brigham Young University, Provo, UT 84602, USA; mtaylo49@byu.edu (M.H.); abigail.kay.johnson@gmail.com (A.C.); scott_weber@byu.edu (K.S.W.)

**Keywords:** Graves’ disease (GD), thyroid-stimulating hormone receptor (TSHR), autoimmune, autoreactive B cell, autoreactive T cell, anti-TSHR autoantibody, thyroid-stimulating antibody (TSAb), hyperthyroidism

## Abstract

Graves’ disease (GD) is a thyroid-specific autoimmune disease with a high prevalence worldwide. The disease is primarily mediated by B cells, which produce autoantibodies against the thyroid-stimulating hormone receptor (TSHR), chronically stimulating it and leading to high levels of thyroid hormones in the body. Interest in characterizing the immune response in GD has motivated many phenotyping studies. The immunophenotype of the cells involved and the interplay between them and their secreted factors are crucial to understanding disease progression and future treatment options. T cell populations are markedly distinct, including increased levels of Th17 and follicular helper T cells (Tfh), while Treg cells appear to be impaired. Some B cells subsets are autoreactive, and anti-TSHR antibodies are the key disease-causing outcome of this interplay. Though some consensus across phenotyping studies will be discussed here, there are also complexities that are yet to be resolved. A better understanding of the immunophenotype of Graves’ disease can lead to improved treatment strategies and novel drug targets.

## 1. Introduction

Graves’ disease (GD) is an autoimmune disease of the thyroid gland. It is one of the most prevalent autoimmune diseases, affecting 20–50 out of 10,000 people every year [1]. It is more frequent in women and those between 30 and 50 years of age. Symptoms of GD are those typical of hyperthyroidism and thyrotoxicosis [2]. Thyrotoxicosis is associated with cardiovascular symptoms, such as tachyarrhythmias, palpitations, angina, mitral valve prolapse, and congestive heart failure [3]. An additional complication is Graves’ ophthalmopathy, which causes inflammation around the eyes and affects about 25–30% of GD patients [4].

**Figure 1:**
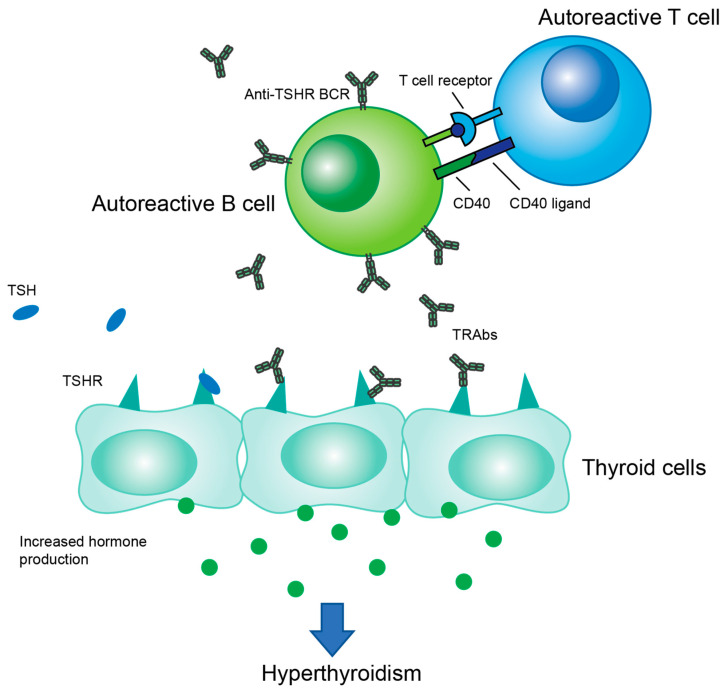
Overview of the immune cell interplay in GD pathogenesis. Autoreactive T cells provide T cell help to autoreactive B cells, which then produce antibodies which bind to TSHR. This antibody binding stimulates hormone production leading to hyperthyroidism.

GD is characterized by an abnormal number of autoreactive B cells that produce antibodies which bind to thyroid-stimulating hormone receptor (TSHR), overstimulating the thyroid and causing hyperthyroidism. These autoreactive B cells and their production of autoantibodies are induced through helper T cell stimulation (Figure 1). The main treatments for GD are antithyroid drugs (ATDs), radioactive iodine therapy (RAI), and in some cases, thyroidectomy [5]. ATDs are generally the first-line treatment, including methimazole, propylthiouracil, and carbimazole. These drugs inhibit the enzyme thyroid peroxidase, leading to a decrease in the production of thyroid hormones. About half of patients relapse into hyperthyroidism after stopping treatment [4]. RAI is usually used after ATDs fail. This treatment attacks the thyroid tissue, slowly reducing it over time. RAI is considered to be a definitive treatment that will eventually result in hypothyroidism and necessitate lifelong hormone replacement therapy. Up to 50% of RAI patients reach hypothyroid state within the first year, with an annual hypothyroid rate of 3–5% thereafter [6]. For severe or persistent cases, thyroidectomy is a fast-acting, effective option. Similar to RAI, it results in hypothyroidism, so lifelong hormone replacement therapy is required post-surgery [4].

As with many autoimmune diseases, the exact root cause of GD is unknown. It is suspected that many factors play a role, including genetics, epigenetics, the gut microbiome, and the imbalance of immune cells [7]. These factors each affect the others, resulting in a complex mechanism that is not yet understood.

Some studies have associated GD with genes that predispose an individual to developing the disease. Some examples are human leukocyte antigen (HLA) genes, cytotoxic T lymphocyte associated 4 (CTLA-4) polymorphisms, and transcription factor forkhead box P3 (FoxP3) polymorphisms [7,8]. Epigenetics has also been explored in GD. DNA methylation, histone modification, and miRNAs are believed to play a role and may cause some of the immunological cell imbalances discussed later [7,9].

The gut microbiome is another factor believed to play a role in autoimmunity. GD patients overall have a lower microbial diversity in the gut compared to normal gut microbiota [10]. Dysbiosis in the gut may affect the proper maturation and differentiation of T cells in the gut-associated lymphoid tissue, leading to alterations in T cell subsets [11,12,13]. Some differences identified in GD patient microbiotas compared to controls are an increase in *Prevotellaceae* and *Lactobacillus* and a decrease in *Bacteroides*, although these alterations differ between geographic regions [7,9,11,12,14].

This review will summarize the research on the topic of cellular phenotyping in GD. We will begin with an overview of the autoantibodies involved in the disease and then discuss the proportions of immunological cell types and subtypes and the role they might play in the pathogenesis of GD with a focus on T cells and B cells.

## 2. Autoantibodies

**Figure 2:**
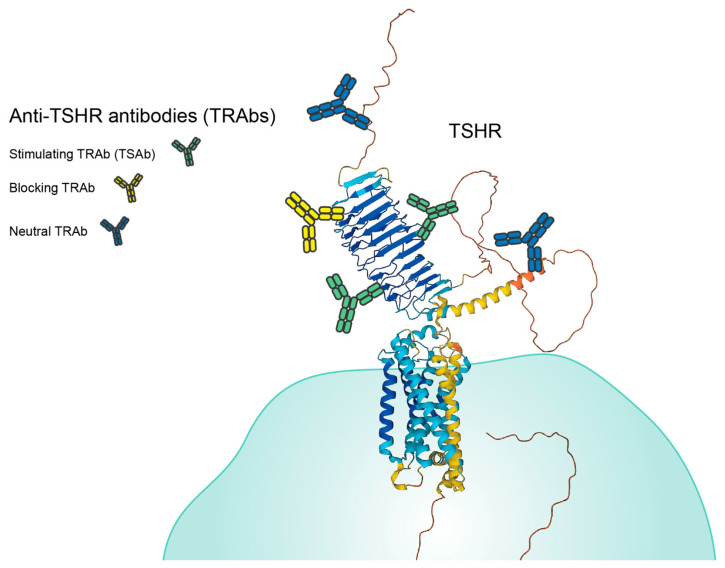
Binding locations of anti-TSHR antibodies (TRAbs). Stimulating TRAbs bind to the leucine-rich repeat domain (LRR), the same domain where TSH binds to its receptor. Blocking TRAbs bind to varied epitopes, the LRR or linear epitopes, but they block TSH from fitting appropriately on the LRR. Neutral TRAbs bind mostly to the linear epitopes of TSHR on the N or C terminal ends. TSHR was modeled using AlphaFold 2™. Antibodies and cells are not to scale.

TSHR autoantibodies (TRAbs) are the major disease-causing agent of GD. There are three categories of TRAbs, TSHR-stimulating (agonist) antibodies (TSAb), TSHR-blocking (antagonist) antibodies, and neutral antibodies; however, TSAbs are typically the most prevalent in a GD patient (Figure 2) [15,16]. The TRAbs that lead to the classical pathogenesis of GD are TSAbs, which mimic the activity of TSH by stimulating the production of two major thyroid hormones, triiodothyronine (T3) and thyroxine (T4). This occurs as the TRAb-binding induces a conformational change in TSHR, signaling the generation of intracellular thyrocyte cAMP, which leads to T3 and T4 production [17]. These stimulating antibodies bind to the leucine-rich repeat (LRR) region of TSHR, the region where TSH binds TSHR as well [18]. The conformation of this region is necessary for both binding to occur and for the conformational change in TSHR [19]. TSAbs are typically IgG class 2 molecules [16]. It has also been shown that epitope spreading can occur during the pathogenesis of the disease in a patient over time as the specific epitopes that the TRAbs bind to changes [20]. It is not yet well understood how epitope spreading affects the pathogenesis of the disease [21].

Blocking TSHR antibodies bind to TSHR in a way that does not stimulate TSHR or cause T3 and T4 production [18,21]. Their binding also prevents TSH from binding to and stimulating TSHR, which can lead to hypothyroidism [18]. The blocking antibodies bind to more varied regions of TSHR, with some binding to the LRR without fitting in the grooves enough to cause a conformational change in TSHR and others binding to the linear epitopes [19,22]. Neutral antibodies also bind to various epitopes of TSHR, though they appear to bind more to the linear epitopes of TSHR [19]. Monoclonal TRAbs have been isolated, produced, and characterized in both agonist and antagonist forms. These have been extensively reviewed by Furmaniak et al. and could have important therapeutic applications in antibody-based treatment (see Section 5) [15].

## 3. T Cells

**Figure 3:**
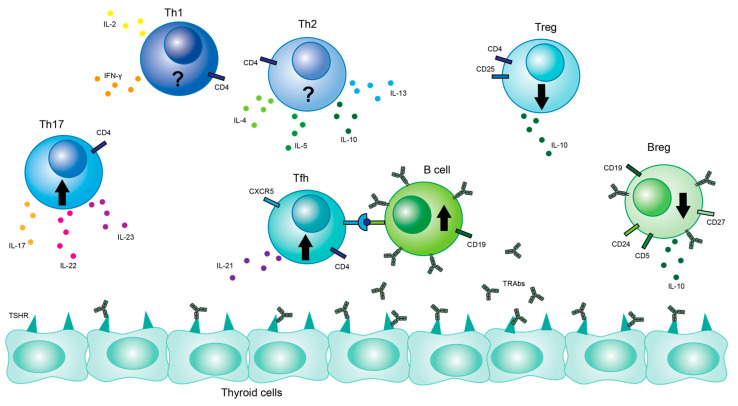
Lymphocyte populations affecting GD. Cell types and their interactions are illustrated. Key cytokines are also pictured. Black up arrows indicate a general increase in the certain cell population in GD patients, while black arrows pointing down indicate a general decrease in the cell population in GD patients. Black question marks indicate that studies on cell population are inconclusive.

After development in the thymus, CD4+ T cells migrate to secondary lymphoid tissue and differentiate into various subsets based on interactions with cytokines and transcription factors [23]. The most well-known of these subsets are Th1, Th2, Th17, and regulatory T cells (Tregs) (Figure 3) [24].

### 3.1. Th17 Cells

Th17 cells are a subset of CD4+ cells that play a role in protecting against infections from bacteria and fungi [25]. The hallmarks of Th17 cells are the production of IL-17A, IL-17F, and IL-22 [23]. There are two subtypes of Th17 cells, pathogenic and nonpathogenic. The pathogenic type secretes pro-inflammatory cytokines and has been associated with various autoimmune diseases, including rheumatoid arthritis (RA) and multiple sclerosis (MS) [25]. The nonpathogenic Th17 cells reside in the gut and help combat harmful bacteria by stimulating the release of antimicrobial peptides. However, these do not cause an inflammatory response against the body [26]. Nonpathogenic Th17 cells secrete IL-10 and pathogenic Th17 cells secrete IL-23 [27].

The majority of studies have found that Th17 cell (CD4+IL17+) levels in the peripheral blood (PB) of newly onset GD patients are elevated compared to controls [28,29,30,31,32,33]. This increase persisted even after ATD treatment [33]. Several studies have shown correlations between the proportion of Th17 cells and TRAb or TSAb activity, suggesting that they may play a role in autoantibody production [28,32]. Similarly, one study also showed a positive correlation between Th17 and T3 and T4 levels, Graves’ recurrent events after therapy (GREAT) scores before treatment, and goiter, suggesting that Th17 cells are related to disease severity [32]. Nevertheless, several studies showed no difference in total Th17 counts between GD and controls, and one study found significantly lower proportions of Th17 in untreated GD patients compared to controls [34,35]. The latter study proposed that the lower levels of Th17 were caused by an influx of lymphocytes to the thyroid tissue that directly decreased peripheral levels [34].

In addition to analyzing cell surface expression by flow cytometry, a variety of other approaches have been used to gauge the prevalence of Th17 in GD. For example, transcriptional levels of ROR-γt, the transcription factor for Th17, were measured and observed to be higher in GD patients compared to controls [29]. Another study found similar results, showing that the mRNA expression of ROR-γt was elevated in GD patients. In the same study, IL-17 mRNA itself was expressed more in GD patients. Th17 was positively correlated with both the IL-17 mRNA levels and the ROR-γt mRNA levels [28]. A different study measured levels of IL-17A mRNA expression and observed no statistical difference between GD patients and controls [36]. However, this finding goes against most of the literature.

### 3.2. Treg Cells

Regulatory T cells have an immunosuppressive function. They downregulate the inflammatory response by four proposed mechanisms, namely inhibitory cytokine release, cytokine deprivation, inhibiting antigen presenting cells, and the direct killing of T cells [37]. Treg cells are closely related to Th17 cells as they stem from the same progenitor cells and are differentiated after induction by TGF-β [23,38,39]. The typical markers that identify Treg cells are CD25 and Foxp3 [40].

An increasing body of evidence shows that Treg cells are either decreased or functionally impaired in GD. Multiple studies found reduced levels of T regulatory cells (CD4+Foxp3+ or CD4+CD25+FoxP3+) in the PB of GD patients [28,29,33,35,41,42]. The surface markers CD25 and Foxp3 were also reduced in PB [43]. However, many other studies have found no statistically significant difference between Treg levels in GD compared to controls [34,44,45,46]. Patients who have undergone ATD therapy have normalized Treg levels [33,35].

One proposed explanation is that although Treg cell levels vary, their function is impaired, contributing to the pathogenesis of GD [44,45]. For example, it was reported that despite comparable levels of Treg cells, the levels of the immunosuppressive cytokine IL-10, one of the primary cytokines that Tregs produce, was significantly lower in GD patients than controls [45]. Similarly, the proliferation rates of effector T cells (CD4+CD25-) were significantly increased when cocultured with Treg cells from GD patients, suggesting that the ability of the Tregs to inhibit proliferation was reduced [46].

Another theory states that although PB levels of Tregs may not be significantly altered, the levels of Tregs in the thyroid tissue itself are reduced [47]. This theory was proposed by researchers who showed that levels of CD4+CD25+Foxp3+ cells were lower in the thyroid tissue than the PB and that the proportion of apoptotic cells was higher among CD4+CD25+ cells in the thyroid than in the PB [47]. This suggests that T regulatory cells may undergo apoptosis when exposed to the microenvironment of the thyroid in GD. Polarized dendritic cells and the unusually high levels of thyroid hormones in the body may create an environment favorable for Treg cells to undergo apoptosis [42]. Further studies are needed to understand the microenvironment of the thyroid and its effect on the infiltrating lymphocytes.

The data on Th17 and Treg cells may be more meaningful when examined together instead of separately. The ratio of Th17/Treg cells is significantly higher in GD than in healthy controls, suggesting that either Th17 cells are increased or Treg cells are reduced or impaired, or some combination of the two [28,29,35]. Few studies have shown that the Th17/Treg ratio is lower in GD than controls [34]. This imbalance in the effects of Th17 and Treg cells is currently believed to play a major role in the pathogenesis of autoimmune thyroid disease [7,23,48].

### 3.3. Th1/Th2 Cells

Th1 and Th2 cells are both subsets of CD4+ T cells with different but related functions. Th1 cells are crucial pro-inflammatory agents against intracellular pathogens. They produce interleukin-2 (IL-2) and interferon-γ (IFN-γ), which help activate CD8+ T cells [23]. Th2 is associated with extracellular pathogens and helps activate B cells and antibody production. They produce the cytokines IL-4, IL-5, IL-10, and IL-13 [7,48]. 

A clear connection has been established between Th1 cells and GD’s counterpart, Hashimoto’s disease, but the results are inconclusive for GD [31]. Studies have shown that abnormal levels of Th1 and Th2 cells are present in GD patients, but other results are contradictory [7]. Since GD is mediated by autoreactive B cells, it has been hypothesized that an increase in Th2 cells plays a role in their development. One study’s findings supported this idea showing that Th2-specific cytokine levels were higher in GD patients than controls. Additionally, the ratio of Th1/Th2 cytokines was smaller, suggesting that the Th2 response was dominant [49]. Innate lymphocyte 2 cells (ILC2s) promote Th2 responses and were also found to be more frequent in GD patients compared to controls [43].

However, Th2 cells also release anti-inflammatory cytokines that may have certain protective functions, suggesting that Th1 cells may be more to blame [23]. A recent study showed that activated Th1 cells are present in higher proportions in patients with newly onset GD than in healthy controls [32]. Another study showed an increase in the Th1/Th2 ratio in GD [35]. Other studies that looked at the levels of Th1 cells and their cytokines in GD patients found no difference between GD patients and controls [30,36].

Additional and more conclusive studies will need to be performed to determine the role of Th1 and Th2 cells in GD. This would help us better understand the pathogenesis of the disease and the effects that potential treatments might have on the immune system. Although a correlation has been observed between Th1 cells and Hashimoto’s disease, a similar connection has not been sufficiently demonstrated in GD itself [23].

### 3.4. Tfh Cells

Follicular helper T (Tfh) cells are found in germinal centers (GC) and play an important role in the specialization of B cells. In the germinal centers, B cells undergo a process of affinity maturation where they fluctuate between the GC’s dark zone, where they proliferate and somatically mutate, and its light zone, where positive selection by Tfh cells favors those B cells with higher affinity to antigen [50,51]. Tfh cells are designated by the expression of chemokine CXC receptor 5 (CXCR5) and often express inducible costimulator (ICOS) or programmed death-1 (PD-1) as well [52]. They can produce large amounts of IL-21, which mediates lymphocyte proliferation and differentiation [53].

Three subsets of Tfh cells can be identified by their expression of chemokine receptor 6 (CCR6) and CXC chemokine receptor 3 (CXCR3): Tfh1 (CXCR3+CCR6-) cells are similar to Th1 cells in that they produce IFN-ỿ; Tfh2 (CXCR3-CCR6-) cells are similar to Th2 cells because they produce IL-4, IL-5, and IL-13; and Tfh17 (CXCR3-CCR6+) cells are similar to Th17 cells because they produce IL-17A and IL-22 [24,54].

Most studies agree that Tfh cells (CD4+CXCR5+ICOS+ or CD4+CXCR5+PD-1+) are significantly elevated in the PB in GD [32,52,55]. Their transcription factor, Bcl-6, may also be elevated [52,56]. According to one study, the main Tfh subset contributing to the elevated levels is Tfh2 cells, which was significantly higher in GD patients both before and after treatment. Tfh1 and Tfh17 were both significantly reduced in GD patients before treatment [55]. These results suggest that Tfh2 cells may be more effective at helping B cells differentiate into autoreactive plasma cells in GD. The study also showed that the levels of circulating Tfh (cTfh) cells in GD patients before treatments were significantly higher compared to healthy controls, while levels of cTfh after treatment were not significantly different than controls [55]. A second study confirmed that the proportion of Tfh cells decreased after treatment with ATD [32]. These researchers hypothesize that the thyroid hormones T3 and T4 may encourage T cell proliferation and the decrease in hormones after treatment correlates to the observed decrease in Tfh cells [32]. One study, however, had different findings, concluding that there was no significant difference in Tfh cells in the PB and that they did not correlate with thyroid hormone levels [57].

Thyroid tissue samples taken from GD patients undergoing thyroidectomy revealed Tfh cells infiltrating the thyroid tissue and the presence of GC-like structures that did not appear in controls [53,58]. These ectopic GCs in the thyroid are similar to normal lymphoid follicles but lack important functional aspects, which could lead to the production of autoreactive B cells [58]. The expression of IL-21 and CXCR5 was also higher in GD thyroid tissue than in the thyroid tissue from controls [53]. IL-21 has been found to be elevated in the serum of GD patients and is strongly correlated with TSAb in untreated patients [52,59]. Tfh cells marked by PD-1 or ICOS are higher in the thyroid tissue than in the PB in GD patients [57].

The dysregulation of T cells in GD is well established. Increased Th17, dysfunctional Tregs, and elevated Tfh cells all seem to play a role. Discovering more about these cell types and their functions in GD will help us better understand the pathogenesis and progression of the disease. 

## 4. B Cells

B cells are an important part of the adaptive immune system, largely because of their ability to mature into plasma cells and produce antibodies. They also present antigens and secrete cytokines. The traditional surface markers for B cells are CD19 and CD20, although CD20 excludes plasma cells [60]. Autoantibodies produced by autoreactive B cells against TSHR are the main pathogenic feature of GD [1].

Researchers have examined the proportions of B cells subsets in GD patients. GD patients appear to have fewer IgM memory B cells (IgM+IgD+CD27+), pre-switched memory B cells (CD27+IgD+), and conventional memory B cells (CD27+IgD−) than healthy controls [32,61]. Conversely, GD patients have more double-negative B cells (IgD−CD27−) and naïve B cells (CD27−IgD+) [32,61]. The proportions of double-negative B cells were also found to be positively correlated with both TRAb and TSAb [32]. Double-negative B cells have been found to be expanded in several other autoimmune diseases, including systemic lupus erythematosus (SLE) and RA, suggesting that they may play a role in autoimmunity [62].

CD11c is an integrin protein usually expressed in dendritic cells. However, CD11c has been found in some B cells and is believed to encourage B cell activation [63]. CD11c+ B cells have been reported as higher in multiple autoimmune diseases [64]. In a study examining GD, Cao et al. found that patients had more CD11c+ B cells than controls, even though the overall number of B cells was not significantly higher [65]. The same study found that these cells (CD11c+) are also positively associated with TRAbs in the PB and that the levels of CD11c+ B cells are higher in the thyroid tissue than the PB. They showed that these cells are poised to be able to turn into antibody-secreting cells and suggested that they may be an important part of the autoreactive B cells that cause hyperthyroidism in GD [65].

Plasma cells (PC) are the subset of B cells that produce antibodies. The frequency of circulating PCs (CD19+CD27^high^CD38^high^) was analyzed by flow cytometry and found to be higher in GD patients than controls [55]. Overall, the percentage of lymphocytes that are B cells are reportedly higher in GD patients than in healthy controls, although the previously cited study by Cao found no difference [61,66]. Similarly, the absolute counts of B cells appear to be higher in GD patients than controls [35]. The correlations between B cells counts and GD need further investigation before concrete conclusions can be drawn.

### Breg Cells

B regulatory cells (Bregs) are distinct from most B cells because of their immunosuppressive function. They secrete IL-10, an anti-inflammatory cytokine which inhibits IL-2 and IFN-γ and downregulates antigen presentation. IL-10 also participates in the downregulation of proinflammatory cells, such as Th17 and Th1 cells [67,68]. Many different subsets of Bregs have been identified, making it hard to define them under a single phenotype, but the surface markers most strongly associated with Bregs are CD5, CD24, CD27, and CD38 [68,69]. CD5 is an important marker on T cells and some B cells that regulate T and B cell receptor signaling. It acts as an immune checkpoint inhibitor and thus could be involved with autoimmune reactions [70]. CD24 and CD27 are thought to be expressed on the surface of cells that produce IL-10 intracellularly [71,72]. CD38 is expressed in many immune cells, especially plasma cells, and it is both an activation marker and an enzyme involved in the production of adenosine [73].

In newly diagnosed patients, the levels of CD19+CD1d^high^CD5+ cells were significantly lower in GD patients than in healthy controls and were negatively associated with TRAb levels, suggesting that they may play a role in inhibiting autoantibody production [28]. CD19+IL-10+ cells were also significantly lower in GD and negatively associated with TRAb levels [43]. Levels of CD19+CD24^high^CD27+ were decreased in newly onset GD, and, additionally, their suppressive capabilities were found to be impaired [72].

Multiple pediatric studies found lower CD19+CD24+CD27+IL-10+ and CD19+IL-10+ cells in GD patients compared to controls [71,74]. The decreased production of IL-10 from CD19+CD24+CD27+ and CD1d+CD5+CD19+ cells was also reported [71]. Possible explanations for the reduced Breg counts and function include Breg exhaustion during autoimmune progression and their migration into the thyroid tissue, making counts in the PB lower [71].

Another pediatric study showed that CD38+Foxp3+IL-10+ cells were decreased in patients compared to controls, but CD38- B cells with either Foxp3 or IL-10 were both increased compared to controls. They propose that the increase in CD38-Foxp3+ and CD38-IL-10+ is a compensatory mechanism used to offset the reduction of the other Breg subsets and counteract the autoimmune reaction [68]. Similar to the impairment of Treg cells, a lack of sufficient or functional Breg cells likely plays a role in the pathogenesis of Graves’ disease based on the studies performed.

## 5. Immunophenotype-Based Treatments

Of the current GD treatments, none can be described as ideal. ATDs have high rates of relapse after withdrawal and RAI and thyroidectomy necessitate lifelong hormone replacement. Given the drawbacks of the current treatments for GD, it is imperative that novel therapies are developed to provide better management of the disease (See summary of novel treatments in Table 1). The immunophenotype of GD patients can be utilized to develop such treatments. 

B cells have been targeted in several small phase I and II clinical trials piloting the drug rituximab for GD [75,76]. Rituximab is a monoclonal anti-CD20 antibody that has been FDA approved for the treatment of non-Hodgkin’s lymphoma and RA. Its use has also been investigated in other autoimmune diseases, including MS and SLE, with positive results [4]. The GD studies saw some response in those with mild disease, but because of small sample sizes and a lack of experimental design features, such as randomization and having a control group, conclusions about the treatment’s effectiveness are limited [4,75,76].

CD40 and its ligand CD154 are part of the signaling pathway that leads to the maturation of B cells [67]. Iscalimab, a blocking anti-CD40 monoclonal antibody, was tested in a fifteen-patient study to see whether inhibiting that pathway ameliorated GD. Three patients responded without relapse within the follow-up period [77]. Although both iscalimab and rituximab did not have high response rates in initial experiments, it is possible that they might improve treatment outcomes when used in combination with ATDs [4,78].

Autoantibody and TSHR peptide-based treatments also show promise in the future treatment of GD [79]. Pearce et al. tested the first antigen specific immunotherapy for GD by synthesizing the soluble synthetic peptide fragments of TSHR (ATX-GD5-9) [80]. These peptides bind with high-affinity to HLA-DR molecules, and their binding without prior internalization reduced the risk of antigen-presenting cell activation. The administration of these peptides to HLA-DR transgenic mice was effective in suppressing T cell and antibody autoimmune response [80]. In a phase I clinical trial, seven out of ten subjects experienced improved symptoms and hormone levels, while the other three displayed worsening thyrotoxicosis [81].

The human monoclonal antibody K1-70™ is a blocking TRAb and is being investigated for therapeutic use [82]. Pre-clinical studies showed that in both rats and monkeys, K1-70™ treatment significantly lowered T3 and T4 levels and increased TSH to normal levels without negative side effects [82,83]. Phase I clinical trials showed that K1-70™ was tolerated at all dosage levels, T3 and T4 levels decreased, TSH levels raised, and clinical symptoms were improved for enrolled GD patients [84]. These immunotherapeutic options based in antibody/antigen specificity show promise for the future treatment of GD.

Aside from current clinical trials, our increasing understanding of the function of immune cells in GD may pave the way for novel treatment approaches, although these concepts remain to be demonstrated. Multiple studies have shown that Th17 proportions are correlated with TRAb levels [28,30,32]. Torimoto et al. also showed a positive correlation between Th17 and T3/T4 levels, GREAT scores before treatment, and goiter [32]. Since Th17 cells appear to be related to disease severity and pathogenesis, they could be a potential therapeutic target or a new prognostic marker [30,32]. Inhibiting their associated cytokines, such as IL-17, may also be an effective treatment, although the effects of systemic IL-17 inhibition may also have many undesirable side effects [37,48].

CD4+ cells have some degree of plasticity, meaning that under the right influences, they can alter their phenotype [48,85]. For example, Treg cells can begin to produce IL-17 in the presence of IL-2, IL-15, IL-21, and IL-23 [23]. This flexibility in effector function means that there is a possibility of developing drugs that promote anti-inflammatory phenotypes in immune cells, restoring balance. If Tregs are truly impaired, as the evidence suggests, providing stimulation that can normalize their function would be an important part of treating GD. On the other hand, if patients are simply Treg-deficient, transfusion of Treg cells from donors could help restore Th17/Treg balance in GD patients [46].

Elevated levels of Tfh cells are associated with GD, as discussed previously. A microRNA has been identified that regulates the Tfh transcription factor Bcl-6. MiRNA-346 was downregulated in GD patients compared to controls and negatively associated with Tfh levels and autoantibody levels. Bcl-6 was found to be overexpressed in the same study [56]. The authors propose that the downregulation of miRNA-346 allows the outgrowth of Tfh cells. Developing a drug that targets miRNA-346 could allow us to manipulate the growth of Tfh cells in GD [56].

Similarly, the IL-21/IL-21R pathway, which is related to Tfh cells, is believed to play a significant role in the GD pathogenesis and production of autoantibodies [53]. The inhibition of IL-21 had an anti-inflammatory effect in RA and SLE models [86,87]. These results suggest that the neutralization of IL-21 may ameliorate disease severity in GD as well [59]. 

GD is a complex disease with many competing factors and currently no satisfactory treatments. We believe that specific antigen-based treatments are likely to be more effective and less harmful than systemic treatments like depleting CD20+ or IL-17+ cells. The further characterization of the cellular and antibody-related interactions in GD will help us define more precise approaches to developing treatments. The research discussed in this paper only scratches the surface of the complex interactions of and the potential treatments for GD.

## 6. Conclusions

In this review we have shown the extent of lymphocyte disproportion in GD as far as it is currently understood. TRAb autoantibodies are characteristic of GD, with the TSAbs as the main cause of symptoms. Epitope mapping has shown that TSAbs mimic TSH and stimulate thyroid hormone production, causing hyperthyroidism. Th17 cells are elevated in GD, as well as Tfh cells. This may lead to increased autoreactive B cell and TRAb levels in GD patients. Treg cells may be reduced or simply functionally impaired, allowing the chronic inflammation and autoimmunity of GD to continue. There is also an important imbalance between Th17 cells and Treg cells which likely influences GD pathogenesis. Double-negative B cells and plasma cells are increased in GD, whereas Breg cells are depleted. This further exacerbates the high levels of autoreactive B cells and TRAbs.

The interplay of immune cells has important ramifications for understanding the cause of GD as well as treating it. More studies are needed to confirm the proportions of immune cells in GD and clarify contradicting claims, such as those about Th1 and Th2 cells. Additional studies are also needed to elucidate the practical significance of the imbalances of immune cells and settle whether they are a causative agent of GD or whether they come about as a result of the disease.

Novel treatment discoveries are underway with the ever-increasing knowledge of GD mechanisms and cell types. The elevated levels of B cells are a relevant target for treatments, with several drugs targeting B cell populations currently in clinical trials. Treatments targeting TSHR peptide/antibody interactions also show promise as highly specific methods of treating GD. Other treatment approaches could be developed as the understanding of the cellular interactions of GD improves.

## Figures and Tables

**Table 1:** Summarizes the therapies based on the immunophenotype of GD in development.

Drug Name	Target	Status	NCT Number
Rituximab	CD20+ B cells	Phase II Trials	NCT00150111
Iscalimab	CD40-CD154 B cell signaling pathway	Phase II Trials	NCT02713256
ATX-GD-59	Activation of APCs	Phase I Trials	NCT02973802
K1-70™	TSHR	Phase I Trials	NCT02904330

## Data Availability

The data for this review are included in the manuscript or the cited papers.
